# Leiomyosarcoma of the Inferior Venacava: A Case Report

**DOI:** 10.31729/jnma.4455

**Published:** 2019-06-30

**Authors:** Diksha Karki, Paricha Upadhyaya, Purbesh Adhikari, Mona Dahal, Bishow Prakash Jung Thapa, Bandana Mudhbari, Gopal Lama, Neera Pathak

**Affiliations:** 1 Department of Pathology, BP Koirala Institute of Health Sciences, Dharan, Nepal

**Keywords:** *inferior venacava*, *leiomyosarcoma*, *smooth muscle actin*

## Abstract

Leiomyosarcoma of inferior venacava is a rare tumor. Female are most commonly affected and middle segment of inferior vena cava is the commonest site. The diagnosis can sometimes be challenging as patients present with non-specific symptoms. We present a case of a 65-year-old female who presented with pain in right hypochondrium and epigastric region since 4 months along with weight loss, anorexia and vomiting. Histopathological examination revealed a capsulated, multilobated tumor arising from muscle layer of inferior venacava with extraluminal growth pattern and tumor cells showing cytoplasmic positivity for immuno-histochemical stain smooth muscle actin. With the diagnosis of leiomyosarcoma of inferior venacava, excision of tumor was done with tangential excision of 4 cm length of inferior venacava with primary repair.

## INTRODUCTION

Vascular Leiomyosarcoma (LMS), arising from smooth muscle of vessel wall, is a rare tumor which constitutes 5-7% of soft tissue sarcomas.^1^ Vascular LMS accounts for 2% of all LMSs with more than half of cases arising from Inferior vena cava (IVC).^2^ Around 75% of cases arise from the retroperitoneal course of IVC.^3^ Apart from IVC, it has also been reported in small vessels like renal, mesenteric, hepatic, saphenous, or gonadal veins.^1^ Due to its rare incidence and unusual clinical course, the early diagnosis and treatment is sometimes challenging.

## CASE REPORT

A 65-year-old female presented with pain in right hypochondrium and epigastric region since 4 months with increase in severity since 15 days with associated weight loss, anorexia and vomiting. On palpation a hard, mobile lump of size 6x8 cm is noted in right hypochondrium. Abdominal Contrast Enhanced Computed Tomography (CECT) scan reveal a heterogeneous mass of size 10x7x8 cm in retroperitoneum compressing IVC, right ureter and right kidney.

Intraoperative finding reveals an encapsulated tumor of size 10x8x8 cm arising from anterolateral wall of infrarenal portion of IVC involving 4 cm length of IVC. With intraoperative diagnosis of IVC LMS, excision of tumor was done with tangential excision of 4 cm length of IVC with primary repair.

**Figure 1.**
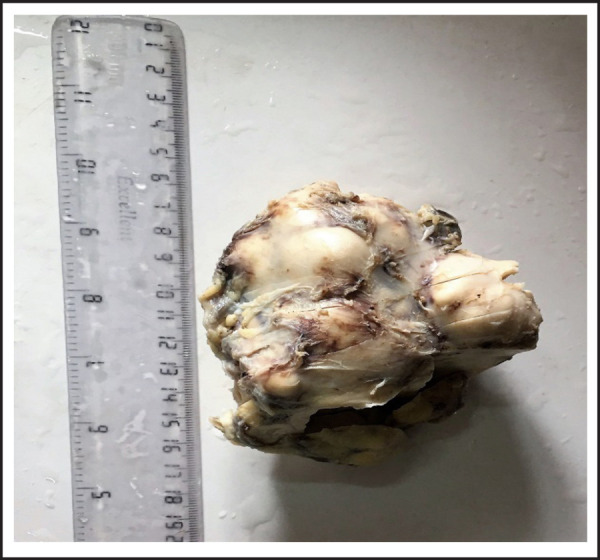
Gross photograph revealed a capsulated, globular mass.

On gross examination, a globular capsulated grey white to grey brown mass (measuring 9x9x5 cm) with attached part of IVC (5x1x0.1 cm) was received (Figure 1).

No capsular breech was identified and resected margins were 0.2 cm away from the tumor. Cut section is solid grey white in color, soft to firm in consistency and multilobated. Areas of necrosis (<50% of tumor) is also identified. Representative sections with resected margins of IVC were submitted for microscopic examination.

**Figure 2.**
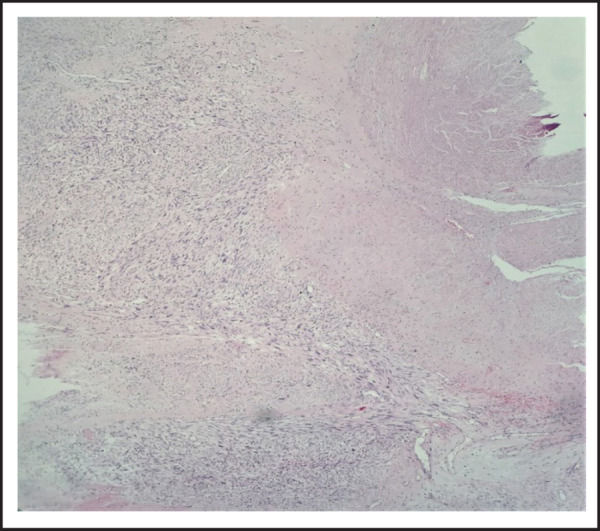
Photomicrograph showing tumor (left) arising from muscle layer of IVC (right); H&E, X40.

After routine tissue processing, Hematoxylin and Eosin (H&E) stained tissue sections were examined under microscope, which revealed an encapsulated, lobulated tumor with intervening septa showing inflammatory cells infiltrate. The tumor is seen arising from the muscle layer of IVC infiltrates through the adventitial layer of vein and is forming an extraluminal mass (Figure 2).

**Figure 3.**
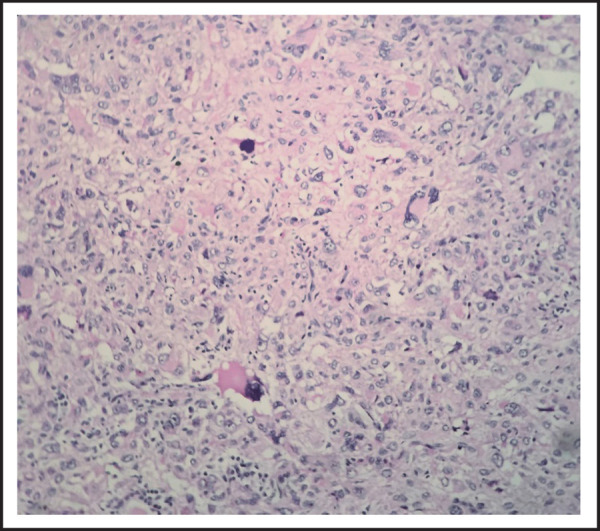
Photomicrograph showing tumor giant cells with few bizarre cells; H&E, X400.

The tumor is composed of intersecting fascicles of moderately pleomorphic spindle cells having eosinophilic cytoplasm and tumor giant cells (Figure 3).

**Figure 4.**
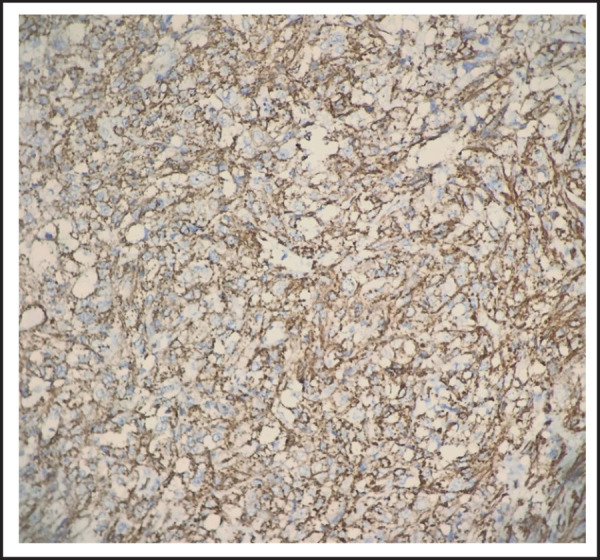
Smooth muscle actin (SMA). Cytoplasmic staining of tumor cells by SMA (DAB x400).

There are areas of necrosis (<50% of tumor) and mitosis (2 per 10 HPF) as well. According to FNCLCC (French Federation of Cancer Centers Sarcoma Group) grading system the tumor was Grade 2.^4^ Resected margins were free and lymphovascular invasion was not identified. Immunohistochemistry showed cytoplasmic staining of tumor cells by Smooth muscle actin (SMA) (Figure 4).

Based on imaging studies, histopathology and immunohistochemistry findings the final diagnosis of Leiomyosarcoma arising from IVC was made.

## DISCUSSION

Vascular LMS is a rare malignant tumor of smooth muscle fiber of tunica media. LMS of IVC was first reported in 1871 in German literature by Perl, since then there are around 400 cases reported.^5^ It is frequently seen in fifth to sixth decade of life with a female predominance.^2,6^ The symptoms depend on its location along the IVC, which can be divided into three segments.^2^ Segment I (lower) is below the renal veins (36% of cases) and present as abdominal pain and palpable mass, Segment II (middle) is from the hepatic veins to the renal veins (44% of cases) and patient present with nephrotic syndrome and Segment III (upper) is from the right atrium to the hepatic veins (20% of cases) and present as Budd-Chiari syndrome.^2,7,8^ In our case tumor was in segment I and patient presented as palpable lump in right hypochondrium.

Hartman et al has described three growth patterns, extraluminal (pattern 1), intraluminal (pattern 2) and both extra and intraluminal (pattern 3) with extraluminal growth pattern being predominantly encountered in cases of retroperitoneal LMS.^9^ When the growth is entirely of pattern 1, LMS has to be differentiated from primary retroperitoneal tumors. The various retroperitoneal tumors like liposarcoma, benign and malignant nerve sheath tumors, myofibroblastic tumor, synovial sarcoma, fibrosarcoma fall into differentials as these tumors can invade the surrounding organs including IVC, during which the primary origin of the tumor can sometimes be difficult to ascertain.^3,10^ However, the presence of interlacing fascicles, eosinophilic cytoplasm, and cigar shaped nuclei in the face of significant atypia can give a clue towards the smooth muscle origin of the tumor. Even the presence of very low mitotic count (<1/10HPF) can suffice the evidence of malignancy.^10^ A similar finding, of low mitotic figure with pleomorphic tumor cells is noted in our case. The direct origin of tumor from muscle of vessel wall with positive smooth muscle actin supported the primary LMS of vascular origin.

Many literatures have described the vascular LMS as having a well circumscribed pseudo capsule. Our case is unique as tumor consists of true capsule and is multilobated.

Imaging modalities such as computed tomography scan, magnetic resonance imaging, ultrasonography aids in determining the origin and extent of the tumor.

Vascular LMS is an aggressive tumor with five year survival noted in around 30 to 50% of the patients who had undergone a complete resection with tumor free margins of 1cm.^8,11^ At present radical resection of the tumor followed by chemotherapy is considered the optimal therapy. In our case total resection of the tumor with vascular reconstruction is done and considering the old age of the patient chemotherapy is not given. The complete resection is possible for those tumors arising in lower segment which is also true in our case where the tumor is completely resectable with a tumor free margin.^8^ Those tumors arising from the middle segment carries a better prognosis.^8^ Following resection around 57% of the cases undergo local recurrence.^2^

LMS can undergo metastasis to various sites like liver, lung, pleura, chest wall, kidney and bone.^7^ No such metastatic lesion was noted in our patient.

In conclusion, vascular LMS is a rare tumor with radical resection of the tumor with microscopic free surgical margin necessary for the overall outcome of the patient.
